# Induction conditions that promote the effect of glycerol on recombinant protein production in *Escherichia coli*

**DOI:** 10.1016/j.btre.2025.e00898

**Published:** 2025-05-16

**Authors:** Yoshihiro Ojima, Hajime Saito, Shintaro Miyuki, Koichi Fukunaga, Terumichi Tsuboi, Masayuki Azuma

**Affiliations:** a Department of Chemistry and Bioengineering, Graduate School of Engineering, Osaka Metropolitan University, 3-3-138, Sugimoto, Sumiyoshi-ku, Osaka 558-8585, Japan; b Sakamoto Yakuhin Kogyo Co., Ltd., 1-2-6, Awaji-machi, Chuo-ku, Osaka 541-0047, Japan

**Keywords:** Glycerol, *E. coli*, Proinsulin production, Catabolite repression

## Abstract

Proinsulin was expressed by *Escherichia coli* SHuffle T7 with pET system in minimal medium containing glucose (Glu medium), glucose and glycerol (GluGly medium) or glycerol (Gly medium). With 100 μM IPTG, proinsulin production did not increase with glycerol. In contrast, proinsulin production per medium volume in GluGly and Gly media was approximately 3∼4-fold higher than in Glu medium with 10 μM IPTG. mRNA expression of target protein was higher in GluGly versus Glu medium, indicating that proinsulin production was enhanced by the release of glucose-induced catabolite inhibition. Although proinsulin production did not differ between GluGly and Gly media at 25 h, substrate was consumed quickly in GluGly medium with 1.55±0.12 times higher proinsulin production at 15 h. Productivity, considering the production period, was highest in the GluGly medium. This study shows a mixture of glucose and glycerol is valuable for protein production in *E. coli* with low IPTG concentrations.

## Highlights

• The recombinant protein production of *Escherichia coli* using pET system was enhanced in glycerol-containing minimal medium with low IPTG concentration.
• Enhanced production in glycerol-containing medium is due to the release of catabolite repression.
• Protein productivity, considering the production period, was highest in the mixture medium of glucose and glycerol.

## 1 Introduction

*E. coli* is the preferable microorganism used for heterologous protein production, accounting for ∼30 % of all recombinant proteins [1,2]. *E. coli* has been used for the large-scale production of proteins because of established genetic tools and a deep understanding of its transcriptional and translational machinery [1,2]. *E. coli* strain BL21(DE3), created by Studier and Moffatt in 1986 [3], is often used in an industrial scale production due to low acetate formation and high replication rates by the integrated T7-polymerase [4,5]. Since the lac operon remains a preferred promotor in pET expression systems [[6], [7], [8]], it is generally used when the target gene is inserted downstream. The repressor protein can be inhibited only by allolactose or a structural analog, such as the typical inducer isopropyl *β*-d-1 thiogalactopyranoside (IPTG) [6].

A favored carbon source used in *E. coli* culture is glucose since this substrate has a very high affinity to the phosphotransferase system [9,10]. Glucose provides a significant amount of energy for cells because it directly enters into glycolysis as glucose 6-phosphate and is consumed through the tricarboxylic acid cycle [10,11]. As a result, the *E. coli* cell growth rate is usually high when using glucose as a carbon source [[12], [13], [14]]. However, culturing *E. coli* that harbors a pET system in a glucose minimal medium causes catabolite repression, which decreases the yield of recombinant protein. Catabolite repression is triggered by the low amount of cyclic adenosine monophosphate (cAMP) in *E. coli* cells at high glucose concentrations [15,16]. This repression is caused by a reduced level of the cAMP receptor protein (CAP)–cAMP complex, which is an activator of the lac promoter. Thus, transcription of the T7 RNA polymerase via the lac promoter in λDE3 lysogens decreases, and the transcription of the target recombinant gene has been shown to decrease [10]. In this way, protein production using the pET system with glucose results in faster cell growth and a lower transcription efficiency of the target protein.

As an alternative carbon source that does not cause catabolite repression, glycerol is promising substrate in terms of biomass yield in *E. coli* cultures [13]. Glycerol was first noticed in biotechnology field as a by-product during biodiesel production [17]. No ccatabolite repression was reported when using glycerol as the primary carbon source in combination with lactose [18]. Although many positive effects of glycerol on recombinant protein production yield in *E. coli* have been reported, glycerol minimal medium causes lower cell growth of *E. coli* and an increase in the culture time [13,14]. Therefore, examining whether a mixture of glucose and glycerol can compensate for each other’s issues in practical protein production is important. Although mixtures of glucose, glycerol and lactose have been shown to increase the production yield of a model protein by *E. coli* as autoinduction systems [19], to our knowledge, no report comprehensively discusses the effects of mixtures of glucose and glycerol on the practical aspects of protein production in minimal media under induction conditions with various IPTG concentrations.

In this study, we aimed to understand the effect of mixed use of glucose and glycerol on recombinant protein production using *E. coli* pET system in minimal medium under different induction conditions. The *E. coli* SHuffle T7 strain was cultured to produce proinsulin, a human insulin precursor, coupled to a monomeric superfolder variant of GFP2 (msGFP2) [20]. Proinsulin is industrially produced using genetically modified *E. coli* and its requirement for diabetes treatment keeps increasing [21]. Proinsulin production was induced using the pET system by adding different IPTG concentrations to cultures growing in minimal media containing glucose, glycerol and a mixture of these two carbon sources. Proinsulin production was evaluated at the transcription and protein expression levels. Substrate consumption and organic acid production were measured, and the results are discussed.

## 2 Materials and methods

### 2.1 Bacterial strains and plasmids

*E. coli* DH5α and SHuffle T7 strains were used (Table 1). SHuffle T7 competent cells were commercially obtained from New England Biolabs (NEB, MA, USA). The pET26b plasmid expressing proinsulin-msGFP2 was constructed by inverse PCR using pQE-60NA-proinsulin-msGFP2 (addgene; 160,466) as the template [20]. The target fragments were amplified with a combination of primers (Table S1). The constructed plasmid was named pET26b-proinsulin-msGFP2 and introduced into *E. coli* SHuffle T7 (Table 1).

**Table 1:** *E. coli* strains and plasmids used in this study.

Strains and plasmids	Note	Reference
*E. coli*		
DH5α	F−, Φ80d *lacZ*ΔM15, Δ(*lacZYA*-*argF*)U169, *deoR, recA*1, *endA*1, *hsdR*17(rK- mK+), *phoA, supE*44, λ−, thi-1, *gyrA*96, *relA*1	General cloning uses
SHuffle® T7	F’ lac,pro,lacI^q^ / Δ(ara-leu)7697 araD139 fhuA2 lacZ::T7 gene1 Δ(phoA)PvuII phoR ahpC* galE (or U) galK λatt::pNEB3-r1-cDsbC (Spec^R^*,lacIq) ΔtrxB rpsL150(StrR) Δgor Δ(malF)3*	New England Biolabs
SHuffle® T7/pET26b-proinsulin-msGFP2	SHuffle® T7 carrying pET26b-proinsulin-msGFP2	This study
Plasmid		
pET26b	T7 RNA polymerase expression plasmid encoding His6 peptide epitope, Kan	Novagen
pQE-60NA-proinsulin-msGFP2	pQE-60NA carrying proinsulin-msGFP2	Addgene ID:160466
pET26b-proinsulin-msGFP2	pET26b carrying proinsulin-msGFP2	This study

### 2.2 Protein production culture

*E. coli* cells were pre-cultured in test tubes containing 4 mL lysogeny broth (LB; 10 g/L Bacto™ Tryptone, 5 g/L yeast extract and 10 g/L NaCl) for 18 h at 37 °C. 50 mg/L kanamycin was added in the culture media for strains carrying pET26b-proinsulin-msGFP2 plasmid. All test cultures were then inoculated into 500 mL baffled conical flasks containing 100 mL minimal medium to an optical density at 660 nm (OD_660_) of 0.2. These were cultured at 37 °C with orbital shaking at 140 strokes/min. The minimal medium contained 3.4 g KH_2_PO_4_, 8.95 g Na_2_HPO_4_･12H_2_O, 2.68 g NH_4_Cl, 0.71 g Na_2_SO_4_, 0.5 g MgSO_4_, 0.067 mg thiamine HCl per liter, and trace metals (2.70 mg FeCl_3_･6H_2_O, 0.59 mg CaCl_2_･2H_2_O, 0.396 mg MnCl_2_･4H_2_O, 0.828 mg ZnSO_4_･7H_2_O, 0.138 mg CoCl_2_･6H_2_O, 0.068 mg CuCl_2_･2H_2_O, 0.096 mg NiCl_2_･6H_2_O, 0.112 mg Na_2_MoO_4_･2H_2_O, 0.070 mg Na_2_SeO_3_, 0.024 mg H_3_BO_3_). Three different carbon sources were used: Glu medium (8 g/L glucose), Gly medium (8 g/L glycerol) and GluGly medium (4 g/L glucose and 4 g/L glycerol). Recombinant proteins were induced by adding IPTG (1, 10, 100 µM) after 3 h culturing. The OD_660_ value was measured as an indicator of cell growth.

### 2.3 Analyses

One milliliter samples were collected from the culture broth to measure glucose and glycerol concentration. After harvesting cells by centrifugation (13,000 × *g*, 5 min, 4 °C), the glucose and glycerol concentrations were determined by high-performance liquid chromatography (HPLC) using an ULTRON PS-80 N column (Shinwa Chemical industries, Ltd., Kyoto, Japan) and ultrapure water as the solvent with a flow rate of 1.0 mL/min at 60 °C. Glucose and glycerol were detected using a refractive index detector. The concentrations of organic acids were also measured by HPLC using an Aminex HPX-87H column (Bio-Rad Laboratories, Inc., CA, USA), with a mobile phase of 20 mM H_2_SO_4_, flow rate of 0.6 mL/min at 55 °C and detection by absorbance at 210 nm.

### 2.4 Sodium dodecyl sulfate-polyacrylamide gel electrophoresis (SDS-PAGE) and western blot

Cells were harvested by centrifugation at 13,000 × *g* and 4 °C for 10 min and resuspended in PBS (pH 7.5). Ten microliter samples of cells were analyzed by SDS-PAGE. Loading samples were prepared on an OD_660_ basis to contain the same quantity of cells. The His-tagged proinsulin-msGFP2 in cells was detected via western blot by transferring the protein from a polyacrylamide gel to an Immobilon-P PVDF membrane (Merck Millipore, MA, USA) using the semi-dry transfer method. Hybridization was conducted using an anti-His-tag primary monoclonal antibody (Medical & Biological Laboratories Co., Nagoya, Japan) and then with a secondary antibody, anti-mouse immunoglobulin G (whole molecule)–alkaline phosphatase (Sigma-Aldrich, MO, USA). Hybridization signals were detected using a BCIP-NBT Solution Kit for Alkaline Phosphatase Stain (Nacalai Tesque, Kyoto, Japan). Target protein levels were quantified by image analysis of the band intensity on the western blot using Image J software (NIH, MD, USA)

At 25 h culturing, the cells were ruptured in PBS by ultrasonication to examine the solubility of the recombinant protein in *E. coli* cells. After centrifugation (13,000 × *g*, 4 °C, 10 min), the supernatant and precipitate were defined as the soluble and insoluble fractions, respectively. The liquid volume of both fractions was adjusted to be equal. These samples were analyzed by SDS-PAGE and western blot.

### 2.5 qRT-PCR

To examine mRNA expression, cells cultured under each condition were harvested at 5 and 10 h culturing by centrifugation at 8000 × *g* and 4 °C for 10 min. Total RNA extraction was conducted as described in our previous study [22] and then reverse-transcribed into cDNA using a PrimeScript RT reagent kit (Takara Bio Inc., Kusatsu, Japan). mRNA expression was analyzed by real-time PCR (CFX Connect, Bio-Rad Laboratories, Inc.), as described previously [23]. The *rrsA* (16S rRNA) was chosen as a reference gene for normalization. The specific primers used are listed in Table S1.

### 2.6 Statistical analysis

All data set for quantitative evaluation were obtained from three independent cultures (biological replicates) in this study. Statistical differences between paired data from two experimental sets were determined with a Student’s *t*-test. To compared paired data from several experimental sets, one way analysis of variance (ANOVA) followed by Tukey test was performed using statistical software (Origin Pro 2022; OriginLab). Values of *p* < 0.05 were considered significant.

## 3 Results and discussion

### 3.1 Proinsulin production with various IPTG concentrations

**Fig. 1:**
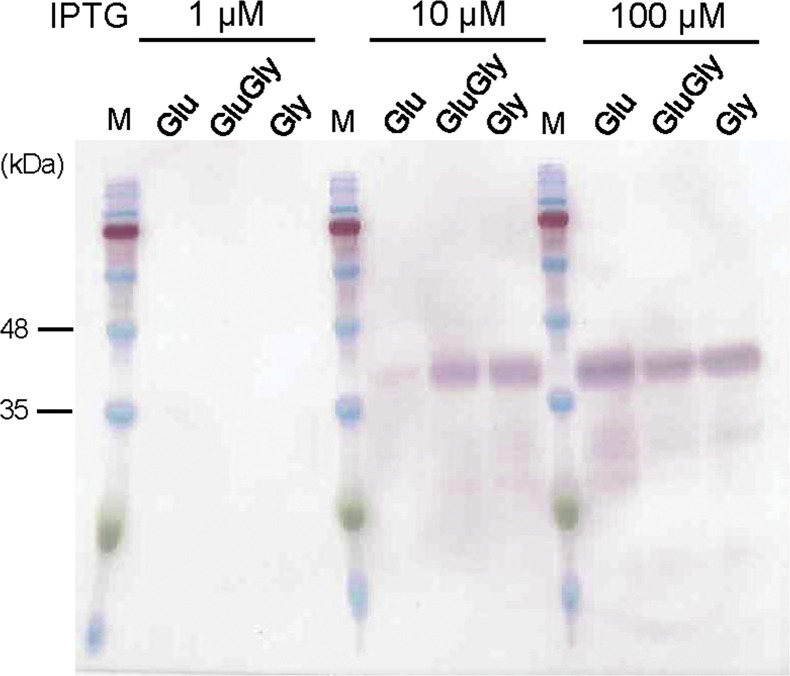
Western blot analysis of proinsulin-msGFP2 in *E. coli* SHuffle T7 cells using the anti-His-tag primary antibody. *E. coli* cells were cultured in a medium containing different carbon sources (Glu, GluGly and Gly media) with various IPTG concentrations (1, 10, 100 µM) for 25 h. The calculated molecular weight of the fusion protein is 37 kDa, which coincides with the bands at a slightly higher position than the 35 kDa marker protein. Loading samples were prepared using the OD_660_ values to ensure that equivalent quantities of cells were used. Therefore, the intensity of each band indicates the amount of proinsulin expressed per cell in each strain.

The expression of proinsulin-msGFP2 by *E. coli* SHuffleT7/pET26b-proinsulin-msGFP2 strain in three types of media (Glu, GluGly and Gly) was compared between various IPTG concentrations (1, 10, 100 µM), as shown in Fig. 1. The maximum concentration of IPTG was set at 100 µM because this concentration was previously defined to be sufficient when glucose and glycerol are used as substrates [14]. Cells were harvested after 25 h culturing, and proinsulin production was analyzed using a western blot with an anti-His tag antibody. Loading samples were prepared on an OD_660_ basis to ensure the same quantities of cells were examined in each sample. In the case of 100 µM IPTG, the bands at a slightly higher position than the 35 kDa marker protein were detected in all three media. The calculated molecular weight of the fusion protein is 37 kDa, suggesting that the proinsulin-msGFP2 was expressed successfully in the constructed strain. The intensity of the bands did not differ noticeably, suggesting that at sufficiently high IPTG concentrations, the expression of the recombinant protein was not reduced by catabolite repression, even in the Glu medium culture.

In the case of 10 µM IPTG, the band intensity in the GluGly and Gly medium cultures was almost the same as those observed when 100 µM IPTG was used. In contrast, the band intensity was very low for the culture grown in the Glu medium, suggesting that the transcription of recombinant protein was repressed in the Glu medium. Thus, protein expression with a lower IPTG concentration (10 µM) was more susceptible to catabolite suppression in the Glu medium. The solubility of the recombinant protein was examined using the cells cultured with 10 µM IPTG (Fig. S1). In SDS-PAGE (Fig. S1A), the protein was confirmed to be present in both the supernatant and precipitate fractions for all three media cultures tested. In contrast, no band was observed in the supernatant fraction from all three media by western blot (Fig. S1B), suggesting that target protein was expressed as an insoluble form. Previous research reported that the fusion tag of small ubiquitin-related modifier (SUMO) supported the partial solubilization of proinsulin when expressed in SHuffle T7 [24]. In the present study, although SHuffle T7 was used as host cell, soluble proinsulin production was not observed due to the lack of fusion tag. On the other hand, it has been reported that insoluble protein production as inclusion bodies generally results in high yields [21]. In this study, we focused on the effects of differences in substrates and culture conditions on total proinsulin production rather than its soluble expression.

When the IPTG concentration was further reduced to 1 µM, no band was detected by western blot under any media condition tested. Previous study also reported that a lower limit of IPTG concentration was 10 μM for protein expression using the pET system [14]. Thus, 10 µM IPTG is effective for protein induction in a minimal medium containing glycerol. Efficient protein production at low IPTG concentrations is important from a production cost viewpoint because IPTG is an expensive additive. Next, we examined the effect of different IPTG concentrations on recombinant protein production in detail.

### 3.2 Proinsulin production with 100 µM IPTG

**Fig. 2:**
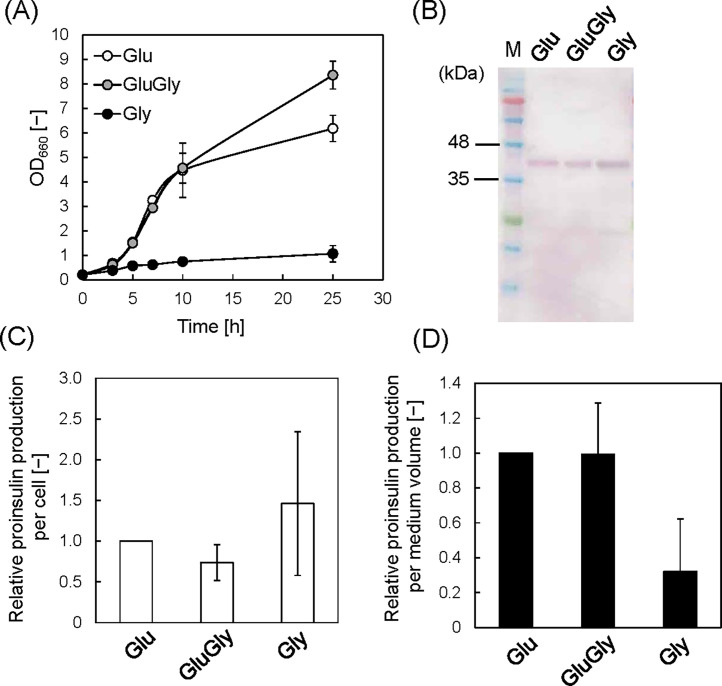
Protein production with 100 μM IPTG in medium containing different carbon sources. (**A**) Growth profiles of *E. coli* SHuffle T7 carrying pET26b-proinsulin-msGFP2. (**B**) Western blot analysis of proinsulin-msGFP2 at 25 h culture using the anti-His-tag primary antibody. Loading samples were prepared using the OD_660_ values to ensure that equivalent quantities of cells were used. (**C**) Relative protein production per cell in each medium. Values were quantified based on image analysis and normalized to that in the Glu medium. Data set was obtained from three independent cultures (*n* = 3). Vertical bars indicate standard deviations. (D) Relative protein production in medium volume by accounting for differences in OD_660_ values.

Fig. 2A shows representative growth curves of the *E. coli* SHuffleT7/pET26b-proinsulin-msGFP2 strain in Glu, GluGly and Gly media. Cell growth in Glu and GluGly media showed similar profiles until 10 h, reaching an OD_660_ of 4.5. Subsequently, while cell growth slowed in the Glu medium, it was maintained in the GluGly medium. The final OD_660_ value at 25 h was 8.4 in the GluGly medium, significantly higher than in the Glu medium (6.2). The enhanced cell growth in a medium of glucose and glycerol was reported previously and rationalized mainly as the low production of acetate [17]. In contrast, the OD_660_ value in the Gly medium was very low throughout the culture period, reaching an OD_660_ of 1.1 at 25 h. The reduced growth rate of *E. coli* in a glycerol medium with a high IPTG concentration was also reported previously [12]. In general, induction with high IPTG concentrations is considered toxic and stresses *E. coli* cells [8,25,26]. Presumably, *E. coli* cells cannot grow normally because of the stress induced by adding IPTG when cultured in medium with glycerol, an energy-poor carbon source.

**Fig. 3:**
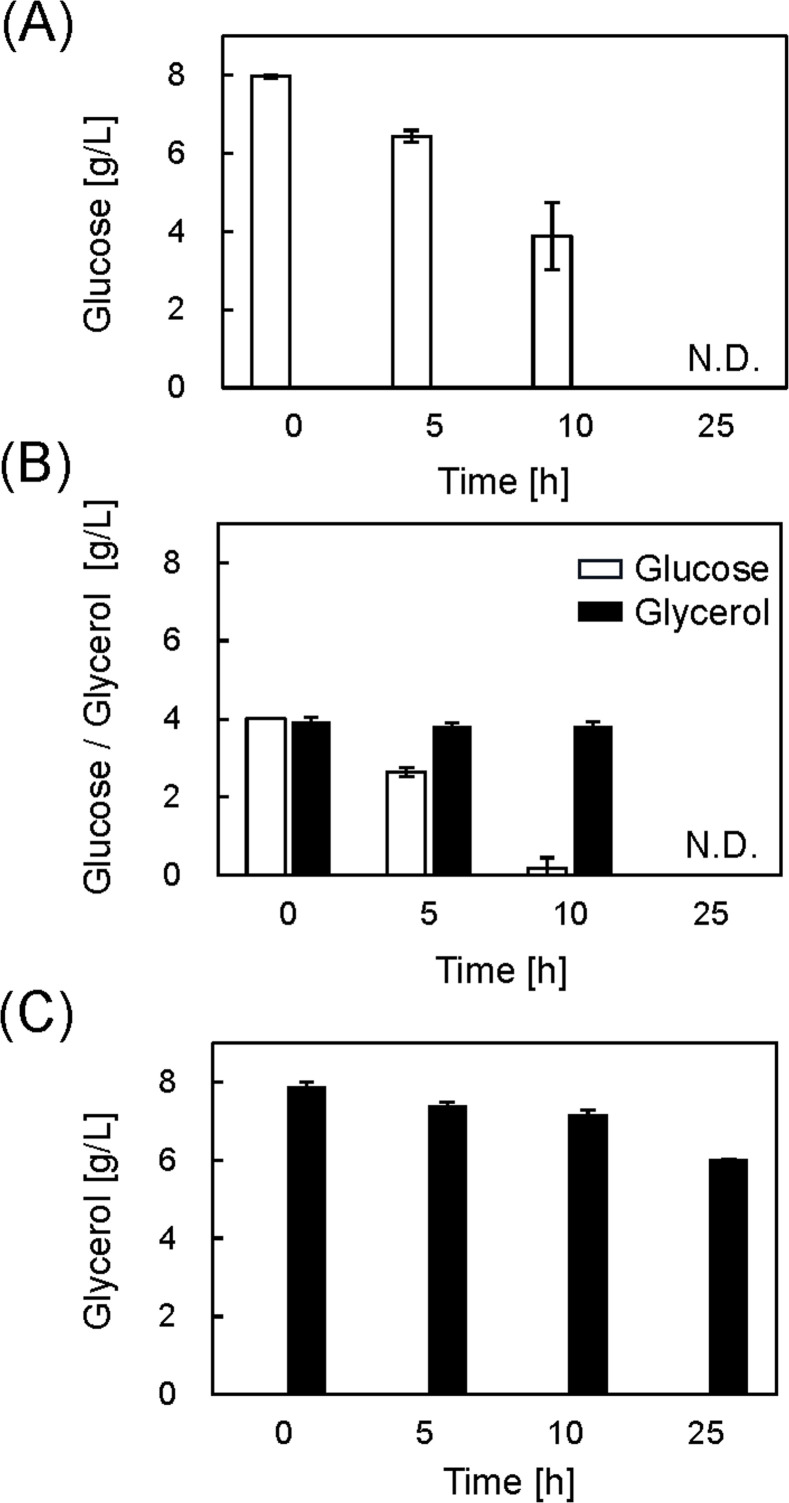
Glucose and glycerol concentrations in each medium with 100 μM IPTG. (A) Glu medium, (B) GluGly medium and (C) Gly medium. N.D.: Not detected. Data set was obtained from three independent cultures (*n* = 3). Vertical bars indicate standard deviations.

The cells were harvested at 25 h, and protein production was analyzed using a western blot with an anti-His tag antibody (Fig. 2B). We further quantified proinsulin production by measuring the band intensities using image analysis (Fig. 2C). The production was normalized to that of the Glu medium result. The relative proinsulin production per cell in GluGly and Gly media was not significantly different from that in the Glu medium. The relative proinsulin production per medium volume was determined by calculating the difference in the OD_660_ values (Fig. 2D). The value in the GluGly medium was almost the same as that in the Glu medium, and the value in the Gly medium was lower. Thus, when the IPTG concentration is sufficiently high, protein production is not improved by using glycerol as a substrate. The glucose and glycerol concentrations were determined by HPLC (Fig. 3). The glucose concentration decreased from 8 to 4 g/L at 10 h for the Glu medium and was completely consumed by 25 h (Fig. 3A). Glucose was essentially consumed after 10 h of culturing in the GluGly medium, whereas glycerol was not consumed until after 10 h (Fig. 3B). Previous research reported that glucose prevents glycerol metabolism because of carbon catabolite repression, and glycerol levels begin to decrease after glucose is completely consumed [17,27,28]. After the glucose level was exhausted at 10 h, glycerol was consumed completely by 25 h. For the Gly medium, the glycerol concentration decreased slightly from 8 to 6 g/L at 25 h (Fig. 3C). This result correlates with low cell growth (Fig. 2A). These results revealed that glycerol is not a suitable substrate when the IPTG concentration is high.

### 3.3 Proinsulin production with 10 µM IPTG

**Fig. 4:**
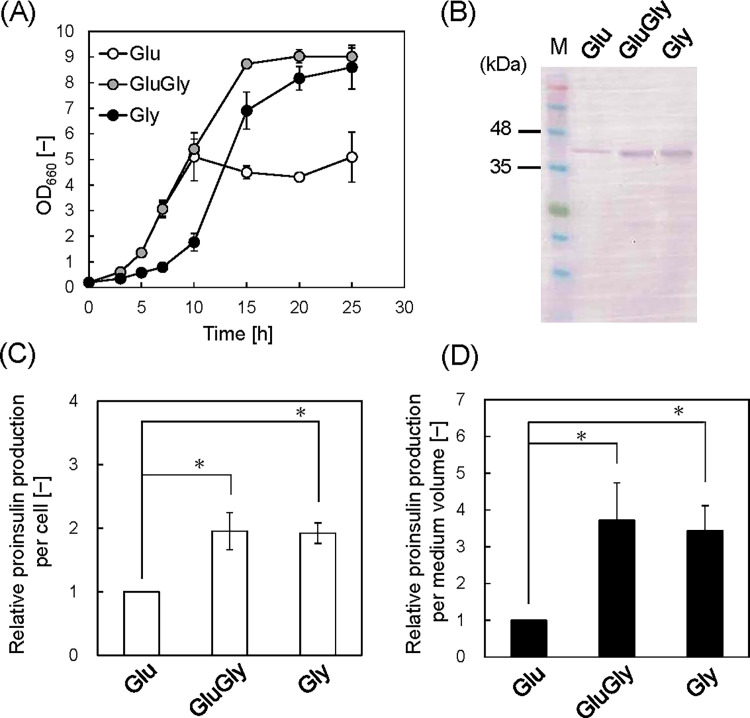
Protein production with 10 μM IPTG in medium containing different carbon sources. (**A**) Growth profiles of *E. coli* SHuffle T7 carrying pET26b-proinsulin-msGFP2. (**B**) Western blot analysis of proinsulin-msGFP2 at 25 h culture. Loading samples were prepared using the OD_660_ values to ensure that equivalent quantities of cells were used. (**C**) Relative protein production per cell in each medium. Values were quantified based on image analysis and normalized to that in the Glu medium. Data set was obtained from three independent cultures (*n* = 3). Vertical bars indicate standard deviations. (D) Relative protein production per medium volume by accounting for differences in OD_660_ values. The statistical significance among the data sets was assessed by ANOVA with the Tukey test (*p < 0.05).

Fig. 4A shows the growth curves in Glu, GluGly and Gly media with 10 µM IPTG. The cell culture in the Glu medium reached an OD_660_ of 5.1 at 10 h, and no appreciable growth was observed thereafter (OD_660_ = 5.1 at 25 h). For the GluGly medium, the OD_660_ reached 5.4 at 10 h, similar to that observed using the Glu medium; however, the cells continued to grow until 15 h, and an OD_660_ of 9.0 at 25 h was attained. This value was slightly higher than that in the GluGly medium with 100 µM IPTG (OD_660_ = 8.4). For the Gly medium, cell growth was delayed and reached an OD_660_ of 1.8 at 10 h and continued proliferation to reach an OD_660_ of 8.6 at 25 h. Thus, lowering the IPTG concentration significantly improved *E. coli* growth using a minimum medium with glycerol as the substrate.

Proinsulin production was analyzed by western blot (Fig. 4B). Loading samples were prepared using the OD_660_ values to ensure that equivalent quantities of cells were used for each media. The relative proinsulin production per cell was quantified by densitometry of the bands using image analysis (Fig. 4C). The relative proinsulin production per cell in GluGly and Gly media was near equivalent, and approximately two-fold higher than that in the Glu medium. These results suggest that the transcription of recombinant protein was repressed by catabolite repression in the Glu medium when the IPTG concentration was 10 µM. The relative proinsulin production per medium volume in GluGly and Gly media was approximately 3∼4-fold higher than in the Glu medium (Fig. 4D). This higher protein production arises from both the higher cell proliferation and higher expression per cell when glycerol is included in the medium.

**Fig. 5:**
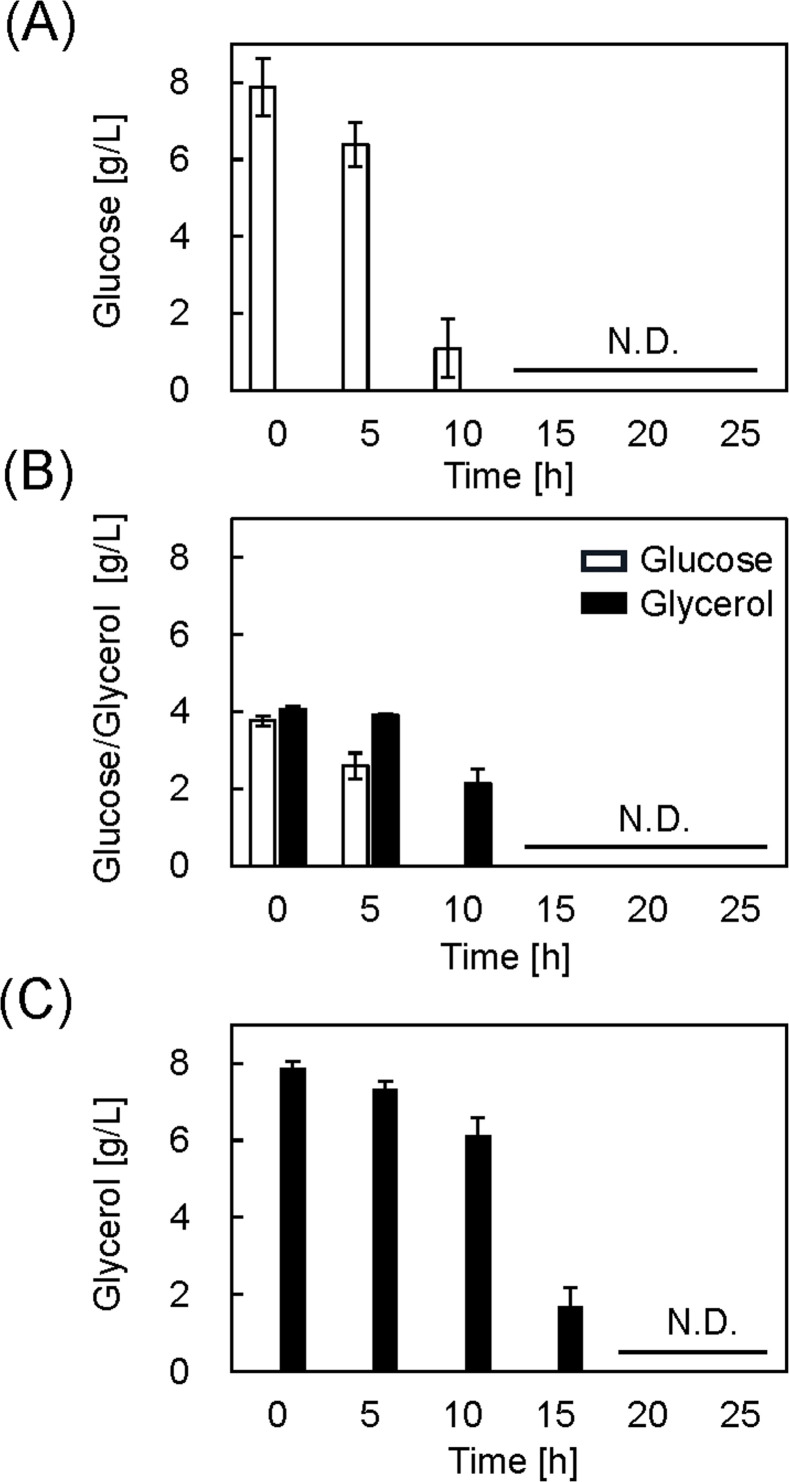
Glucose and glycerol concentrations in each medium with 10 μM IPTG. (A) Glu medium, (B) GluGly medium and (C) Gly medium. N.D.: Not detected. Data set was obtained from three independent cultures (*n* = 3). Vertical bars indicate standard deviations.

Because proinsulin production per medium was equal in GluGly and Gly media, the productivity was compared by considering the production period. Substrate concentrations were measured by HPLC to determine the culture end point (Fig. 5). In the case of Glu medium, glucose drastically decreased from 5 to 10 h, and the remaining glucose was completely consumed from 10 to 15 h (Fig. 5A). Glucose was consumed completely from 5 to 10 h in the GluGly medium, and glycerol consumption started at 10 h (Fig. 5B). Glycerol was consumed completely by 15 h in GluGly medium. On the other hand, glycerol slowly decreased by10 h in the Gly medium and remained at 15 h (Fig. 5C). Glycerol was consumed completely by 20 h in Gly medium. These results suggest that the production period is shorter in the GluGly medium. To evaluate the productivity, proinsulin production at 15 h was analyzed using western blot (Fig. S2A). The intensity of the bands did not significantly differ between GluGly and Gly medium (Fig. S2B). However, the OD_660_ in GluGly medium at 15 h was 8.7 and higher than that in Gly medium (6.9). Therefore, the calculated relative proinsulin production per medium volume considering the OD_660_ value at 15 h was significantly higher (1.55 ± 0.12 times) in GluGly medium compared to that in Gly medium (Fig. S2C), suggesting that the proinsulin production was completed just after glycerol runs out (15 h) in the GluGly medium. From these results, we conclude that the productivity, considering the production period, was highest in the GluGly medium for recombinant protein production using *E. coli* with a pET system.

### 3.4 Mechanism for enhanced growth and proinsulin production with glycerol and 10 µM IPTG

**Fig. 6:**
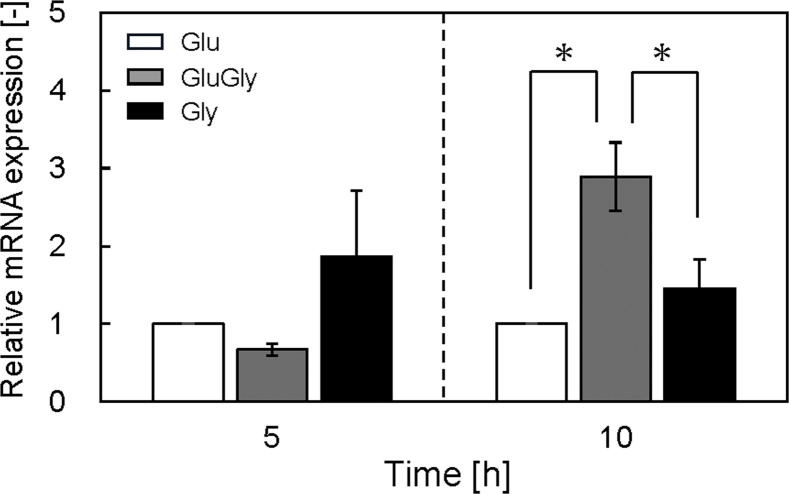
Relative mRNA expression of proinsulin-msGFP2 cultured in each medium. mRNA expression was analyzed by qRT-PCR. Data set was obtained from three independent cultures (*n* = 3). The statistical significance among the data sets was assessed by ANOVA with the Tukey test (*p < 0.05).

Ten micromolar IPTG was suitable for efficient protein production in a minimal medium containing glycerol using the *E. coli* T7 expression system. Next, the mRNA expression of the recombinant protein was analyzed to support the hypothesis that the increase in protein production was achieved by alleviating catabolite repression. Based on the time course of OD_660_ and substrate concentrations (Fig. 5), mRNA expression was evaluated at the growth phase (5 and 10 h). Fig. 6 shows the relative mRNA expression of proinsulin-msGFP2. At 5 h, the mRNA expression level in the GluGly medium was not significantly different from that in the Glu medium. Residual glucose in the GluGly medium at 5 h suppressed expression because of catabolite repression. In contrast, expression in the Gly medium was generally higher than in the Glu or GluGly medium. At 10 h, the mRNA expression in the GluGly medium was three-fold higher than that in the Glu medium. Because glucose in the GluGly medium was completely consumed from 5 to 10 h, catabolite suppression was released by switching the substrate to glycerol, increasing protein expression. These results suggest that the high amount of proinsulin per cell in the GluGly medium was because of the high transcription level.

Organic acid production was analyzed by HPLC to determine why growth is promoted in a medium containing glycerol (Fig. S3). For the Glu medium, acetate, citrate and α-ketoglutarate were mainly produced at a maximum of ∼1 g/L (Fig. S3A). In particular, acetate remained in the medium even at 25 h. The concentration of organic acids in the GluGly medium was very low throughout the culture period (Fig. S3B). Citrate was detected in the Gly medium; however, this metabolite had been consumed by the end of the culture period (Fig. S3C). Thus, promoted cell growth in GluGly or Gly medium was caused by the lower conversion of the substrate to organic acids. A previous paper reported that an increased flux through the TCA cycle, glyoxylate shunt, acetate consumption and gluconeogenesis occurs when *E. coli* BL21 cells are grown in glycerol as the sole carbon source, which may explain the lower carbon loss and acetate overflow [13]. In this study, organic acid production was completely suppressed using a mixture of glucose and glycerol.

In conclusion, proinsulin production per cell in GluGly and Gly media was approximately two-fold higher than in Glu medium when IPTG was reduced from 100 to 10 μM. Furthermore, cell growth was promoted in media containing glycerol, and proinsulin production per medium volume in GluGly and Gly media was approximately 3∼4-fold higher than that observed in the Glu medium. In the GluGly medium, mRNA expression was higher than in the Glu medium after 10 h culturing, indicating that proinsulin production was enhanced because of the release of glucose-induced catabolite inhibition. Glycerol was consumed faster in the GluGly medium than in the Gly medium with increased protein production, suggesting that the productivity, considering the production period, was highest in the GluGly medium.

## CRediT authorship contribution statement

**Yoshihiro Ojima:** Writing – original draft, Validation, Supervision, Resources, Project administration, Methodology, Investigation, Formal analysis, Data curation, Conceptualization. **Hajime Saito:** Writing – review & editing, Validation, Methodology, Investigation, Formal analysis, Data curation. **Shintaro Miyuki:** Writing – review & editing, Project administration, Methodology, Investigation, Data curation. **Koichi Fukunaga:** Writing – review & editing, Validation, Project administration, Methodology, Investigation, Formal analysis, Data curation. **Terumichi Tsuboi:** Writing – review & editing, Supervision, Project administration, Formal analysis, Data curation, Conceptualization. **Masayuki Azuma:** Writing – review & editing, Validation, Supervision, Methodology, Formal analysis, Data curation.

## Declaration of competing interest

There is no conflict of interest to declare regarding our paper entitled “Induction conditions that promote the effect of glycerol on recombinant protein production in *E. coli*”.

## Data Availability

Data will be made available on request.

## Appendix Supplementary materials

Image, application 1

Image, application 2
