# Supplementary material for: Induction conditions that promote the effect of glycerol on recombinant protein production in Escherichia coli
Source: Biotechnol Rep (Amst). 2025 May 16;46:e00898. doi: 10.1016/j.btre.2025.e00898 (PMC12152334; doi:10.1016/j.btre.2025.e00898)
Supplement: Supplementary file 1 [file mmc1.docx]

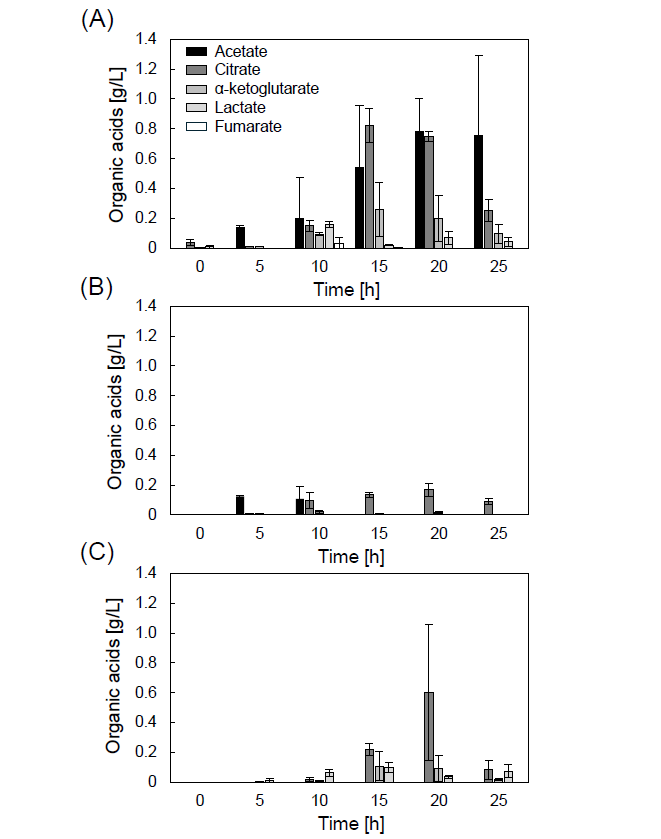


Fig. S1 Solubility analysis of proinsulin-msGFP2 expressed in *E. coli* SHuffle T7 cells cultured in medium containing different carbon sources with 10 μM IPTG. The cells cultured for 25 h were harvested and ruptured by ultrasonication. After centrifugation, the supernatant (Sup) and precipitate (Ppt) were analyzed by (A) SDS-PAGE and (B) Western blot using the anti-His-tag primary antibody.


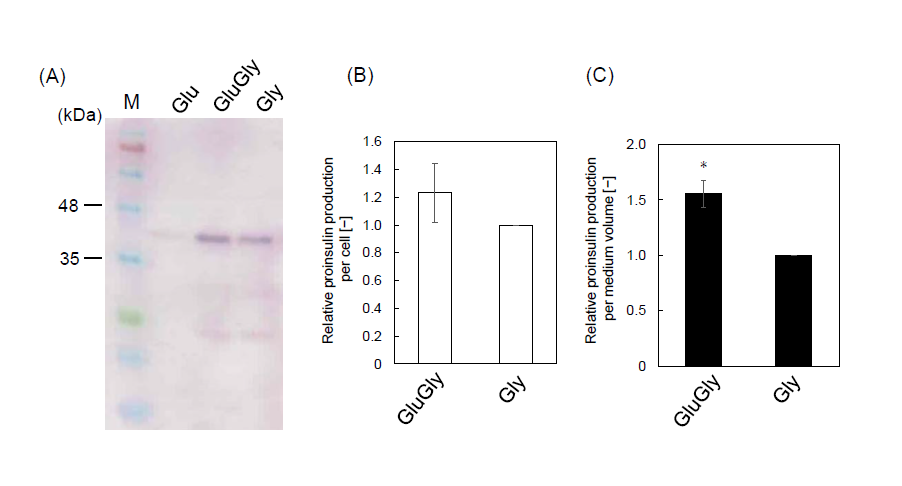


Fig. S2 Protein production with 10 μM IPTG at 15 h in medium containing different carbon sources. (**A**) Western blot analysis of proinsulin-msGFP2 at 15 h culture. (**B**) Relative protein production per cell in each medium. Values were quantified based on image analysis and normalized to that in the Gly medium. (C) Relative protein production per medium volume by accounting for differences in OD_660_ values. Data set was obtained from three independent cultures (n=3). Vertical bars indicate standard deviations. The statistical significance among the data sets was assessed by the Student’s *t*-test (**p* < 0.05).


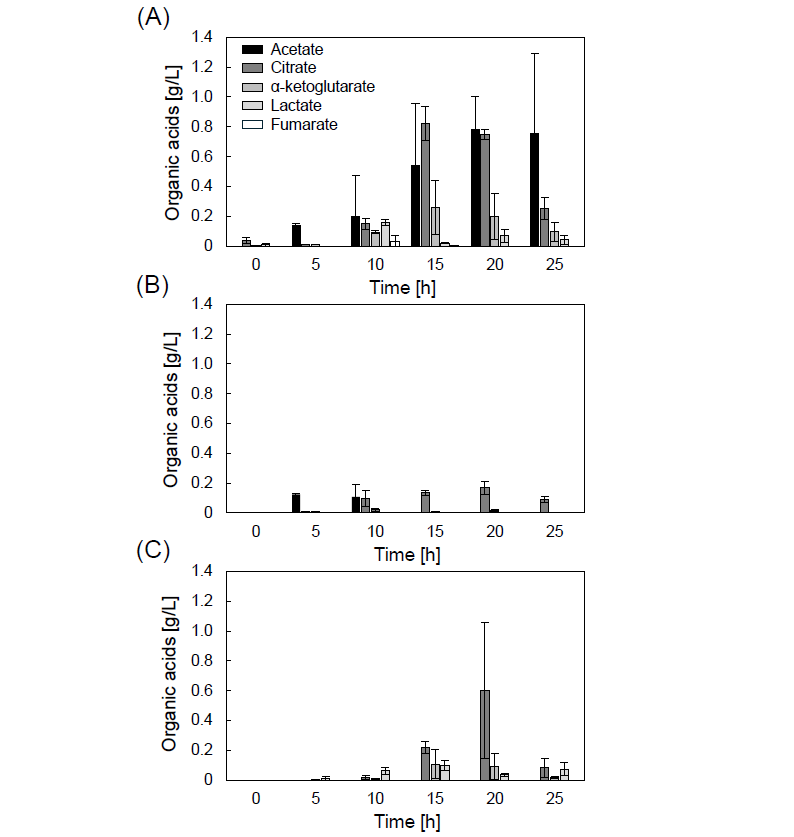


Fig. S3 Organic acid concentrations in each medium with 10 μM IPTG. (A) Glu medium, (B) GluGly medium and (C) Gly medium. Data set was obtained from three independent cultures (n=3). Vertical bars indicate standard deviations.
