# Supplementary material for: Induction conditions that promote the effect of glycerol on recombinant protein production in Escherichia coli
Source: Biotechnol Rep (Amst). 2025 May 16;46:e00898. doi: 10.1016/j.btre.2025.e00898 (PMC12152334; doi:10.1016/j.btre.2025.e00898)
Supplement: Supplementary file 2 [file mmc2.docx]

| Supplementary Table S1 Primers used in this study | |
| --- | --- |
| Primers | Sequences |
| Inverse PCR for pET26b-proinsulin-msGFP2 |  |
| *pET26b* -F | 5′- TAAGCTTGCGGCCGCACTCGAGCACCACCACCAC -3′ |
| *pET26b* -R | 5′- TAAACAAAATTATTTCTAGAGGGGAATTGTTATCCGCTC -3′ |
| *proinuslin*-F | 5′- AAATAATTTTGTTTAACTTTAAGAAGGAGATATACATATGTTTGTGAACCAACACCTGTGCG -3′ |
| *proinsulin* -R | 5′- GCGGCCGCAAGCTTAGAACCGCCACTGCCTCCAC -3′ |
| Sequencing primers |  |
| F | 5′- TCCCGCGAAATTAATACGAC -3′ |
| R | 5′- CCCTCAAGACCCGTTTAGAG -3′ |
| qRT-pPCR primers |  |
| *rrsA*-F (internal reference) | 5′- TACGACCAGGGCTACACACG -3′ |
| *rrsA*-R (internal reference) | 5′- ATCCGGACTACGACGCACTT -3′ |
| *proinsulin*-msGFP2-F | 5′- AAGGGCATCGACTTCAAGGA -3′ |
| *proinsulin*-msGFP2-R | 5′- TGTCGGCGGTGATATAGACG -3′ |
